# Effectiveness of a guided digital self-help intervention to improve sleep and the biological clock in university students – Study protocol for a randomized controlled trial

**DOI:** 10.1016/j.invent.2024.100763

**Published:** 2024-08-02

**Authors:** Laura M. Pape, Annemieke van Straten, Sascha Y. Struijs, Philip Spinhoven, Niki Antypa

**Affiliations:** a Department of Clinical Psychology, Leiden University, the Netherlands; b Department of Clinical, Neuro- and Developmental Psychology, VU University Amsterdam, the Netherlands; c Amsterdam Public Health Research Institute, Vrije Universiteit Amsterdam, the Netherlands

**Keywords:** E-health, Intervention, Sleep, Biological clock, Self-help, CBT-I, University students

## Abstract

**Background:**

Sleep problems occur in many university students which affects their mental health and daily functioning. Cognitive behavioural therapy for insomnia (CBT-I) has been proven effective in adults but research in university students, who struggle to maintain a 24-hour rhythm, is still limited. We hypothesize that a guided digital CBT-I intervention, enriched with components on the biological clock (*‘i-Sleep & BioClock’*) will be effective in reducing insomnia severity and improving mental health outcomes for students with sleep problems.

**Objectives:**

We aim to evaluate the effectiveness of a guided online sleep and biological clock self-help intervention in improving sleep, depression symptoms, anxiety symptoms, functioning, academic performance, and quality of life in university students at 6 weeks and 18 weeks.

**Methods:**

This is a two-arm parallel-group superiority randomized controlled trial, comparing a 5-week guided online *‘i-Sleep & BioClock’* intervention to online psychoeducation (PE). We aim to include 192 university students (Bachelor, Master, and PhD) with at least subthreshold insomnia (Insomnia Severity Index ≥10), aged ≥16, who can speak Dutch or English. We are excluding students with current risk for suicide or night shifts. The primary outcome is insomnia severity. Secondary outcomes include sleep estimates (sleep and light exposure diary), depression, anxiety, functioning, quality of life, and academic performance. The effectiveness of the intervention compared to online PE will be evaluated using linear mixed models.

**Discussion:**

The current study tests the effectiveness of an online self-help intervention for university students who suffer from sleep problems. This trial builds upon an open feasibility study and will provide evidence of an online guided self-help program for students. The findings of this study will determine the potential wider dissemination of the intervention to address the high need for available and accessible help for students experiencing insomnia.

**Trial registration:**

ClinicalTrials.Gov (NCT06023693), registered on August 3rd, 2023.

## Highlights

• This protocol describes the study design of a randomized controlled trial.
• We will compare a guided digital self-help intervention to online psychoeducation.
• We aim to include 192 university students with clinically significant insomnia.
• The outcomes include insomnia severity (primary outcome) and mental health outcomes.

## 1 Background

Sleep problems are very common among university students. The prevalence of insomnia among university students is higher than in the general adult population, with 18.5 % of students suffering from insomnia compared to 7.4 % in the general population (Jiang et al., 2015). In addition, less severe forms of sleep deprivation are common among students as well, with 70.6 % of students obtaining less than the recommended 8 h of sleep per night, disrupting their circadian rhythm (Lund et al., 2010). In a recent study at Leiden University, about 40 % of a sample of 742 students suffered from poor sleep quality, and about 36 % suffered from depressive symptoms, meeting criteria above the clinical threshold (Van Den Berg et al., 2018).

Reasons for sleep problems among students can be high levels of stress, performance pressure, irregular academic and social schedules, late-night use of electronic devices, caffeine, alcohol, and drug use (Lund et al., 2010; Taylor Daniel, 2012; Hershner and Chervin, 2014). The different demands and conflicting priorities faced by students can make it much more difficult for them to maintain a 24-hour rhythm and can lead to irregular sleep-wake patterns, which might be one of the key factors that set this group apart from young adults in general and other populations (Lund et al., 2010; Hershner and Chervin, 2014). The consequences of persisting sleep disturbances in terms of student's mental health, daily functioning, and academic performance are severe (Hershner and Chervin, 2014; Kayaba et al., 2020). For instance, insomnia has been associated with a higher risk of failed examinations and delayed study progress (Vedaa et al., 2019), and chronic sleep deprivation is a predictor for lower academic achievements (van der Heijden et al., 2018). These struggles in educational success can lead to direct and indirect costs for the individual and to society as a whole, and add additional stress and pressure to a student's life.

Mental health problems in students can be caused by a myriad of factors, among which sleep problems seem prominent. Recent systematic reviews have confirmed strong associations of insomnia with depression and anxiety (Pigeon et al., 2017; Baglioni et al., 2011; Suh et al., 2013; Hertenstein et al., 2019). Longitudinal meta-analyses have found that insomnia is a predictor for depression and anxiety with a two- to three-fold risk (Baglioni et al., 2011; Hertenstein et al., 2019). When untreated, persistent insomnia also significantly increases the risk of suicidal ideation (Suh et al., 2013). University students are an especially vulnerable group as their age group is the most common for the onset of several mental health disorders, which is why early preventative interventions are crucial in this population (Zivin et al., 2009; Kessler et al., 2005).

Psychological interventions aimed at insomnia are not only effective in reducing insomnia and its related burden itself but also offer a less stigmatized way to improve mental health outcomes versus offering interventions addressing issues such as depression or anxiety (Cuijpers, 2021). The most common used treatment for insomnia is Cognitive Behavioural Therapy for Insomnia (CBT-I) (Riemann et al., 2017). CBT-I is a treatment designed to break patterns of maladaptive thinking and behaviour that are common in insomnia, and typically includes behavioural, cognitive, and educational components. Several studies have shown that CBT-I interventions are effective in not only reducing insomnia symptoms, but also improving mental health outcomes such as depression, and sleep-related quality of life in adults reporting insomnia symptoms (van der Zweerde et al., 2019a; Espie et al., 2019; Christensen et al., 2016). The current body of evidence suggests that interventions targeting sleep have broader positive implications on mental health, such as anxiety and depression (Kodsi et al., 2022), and even paranoia and psychotic experiences (Freeman et al., 2017). Although CBT-I interventions have been proven effective in adults (Van Straten et al., 2018), research in university students is still somewhat limited. Meta-analyses summarizing the evidence on university students include different types of psychological sleep interventions (Saruhanjan et al., 2021a; Friedrich and Schlarb, 2018; Chandler et al., 2022) and included few studies on CBT-I interventions (Taylor et al., 2014; Baroni et al., 2018) and few that were delivered digitally (Freeman et al., 2017; Morris et al., 2016; Trockel et al., 2011; Asano et al., 2015; Denis et al., 2020). Recent literature suggests that students might even benefit more from psychological interventions, especially CTB approaches, than other populations due to their high cognitive flexibility and greater potential to change behaviour (Friedrich and Schlarb, 2018; Saruhanjan et al., 2021b).

*E*-health and computer-based interventions have become increasingly popular during the last years. Especially the COVID-19 pandemic created a turning point for online therapy (Wind et al., 2020; Van De Vijver et al., 2023). E-health interventions are a great opportunity for university students, who are often hesitant to reach out to professional mental health services, since they either tend to seek rather informal help or prefer to solve their problems on their own (Blanco et al., 2008; Struijs, 2023). The advantages of e-health programs are that they are cost-effective, easily accessible, can be completed at the participant's own pace, and can be used anonymously which creates a more comfortable environment for sensitive topics such as mental health issues (Ryan et al., 2010; Davies et al., 2014). Meta-analyses of digital CBT-I interventions in the adult population have shown positive and long-lasting effects on subjective sleep quality, insomnia severity, and depression (Seyffert et al., 2016; Zachariae et al., 2016).

The life of a university student typically includes irregular sleep-wake schedules due to late-night-studying, social activities, part-time jobs, and other factors. Oftentimes, students struggle to maintain a 24-hour rhythm, which plays a key role in establishing a healthy sleep pattern. Dysfunctional circadian rhythms can desynchronize the sleep-generating process and can lead to fragmented or insufficient sleep, as well as contribute to sleep onset insomnia when the timing of this rhythm is too late or early morning awakening insomnia when the timing is too early (Lack et al., 2017). Young adults are on average more prone to eveningness meaning they have a natural tendency to go to bed late and wake up late (Roenneberg et al., 2007). In several student samples, the evening chronotype, which is the natural preference to fall asleep and wake up late, has been associated with poorer sleep quality (Henrich et al., 2023), higher depressive symptoms (Van Den Berg et al., 2018), and lower mental health-related quality of life (Kayaba et al., 2021). The mismatch between societal demands and students' late circadian preference can further maintain their sleep problems, by shortening the sleep duration during the week and increasing social jetlag, which is a type of circadian misalignment characterized by the discrepancy between an individual's sleep pattern on work/study days versus free days (Roenneberg et al., 2019).

Therefore, by learning more about circadian rhythms and understanding their own biological clock, students can improve their sleep and overall wellbeing by adjusting their sleep patterns. Most CBT-I interventions to date have not taken into account the biological clock, meaning that less attention has been given to the importance of light exposure, differences in chronotypes, and other aspects common in university students such as social jetlag. This study aimed to add these components in order to enhance the effectiveness of the existing CBT-I intervention protocol. Considering the known effectiveness of CBT-I interventions and the role of circadian rhythmicity in students, we developed a guided e-health intervention (‘*i*-*Sleep & BioClock*’) designed to improve sleep and regulate the biological clock, eventually aiming to reduce broader mental health problems in university students.

### 1.1 Aims and hypotheses

The objective of this study is to evaluate the effectiveness of the guided online ‘*i*-*Sleep & BioClock*’ intervention in reducing insomnia in university students compared to an online psychoeducation control program. We hypothesize that the intervention group will show significantly more improvement in insomnia severity (primary outcome) than the control group at post-test, which will be maintained at 18-week follow-up. Similarly, we expect significant improvements in secondary outcomes assessed by the sleep & light exposure diary, depression and anxiety symptoms, functioning, academic performance, and quality of life. Since young adults are more prone to eveningness (Roenneberg et al., 2007), we expect that chronotype will on average shift toward morningness. Additionally, social jetlag is expected to decrease in both groups since fixed bedtimes are recommended.

The secondary aim of this study is to explore potential mediators of the intervention effect. Cognitive mediators, such as pre-sleep arousal and dysfunctional beliefs and attitudes about sleep were found to be mediators of the effect of CBT-I interventions in the adult population, which is why we hypothesize that they will be mediators in the student population too (Lancee et al., 2019; Parsons et al., 2021). Moreover, light exposure, circadian shift, and variability in sleep patterns will be examined as behavioural mediators (Schwartz and Carney, 2012).

The third aim of the study is to investigate potential moderators of treatment response, in order to determine who benefits most of the intervention. We hypothesize that chronotype influences the responsiveness to CBT-I, but not to online psychoeducation since the *‘i-Sleep & BioClock’* intervention includes intervention elements specifically targeting the biological clock. Other variables, such as sociodemographic variables and symptom severity, will also be tested as moderators, using tree-based interaction models. Outcome expectations, adherence to the program, and stressful life events are expected to be predictors of treatment response (Constantino et al., 2018; Li et al., 2019).

## 2 Methods

### 2.1 Study design

**Fig. 1:**
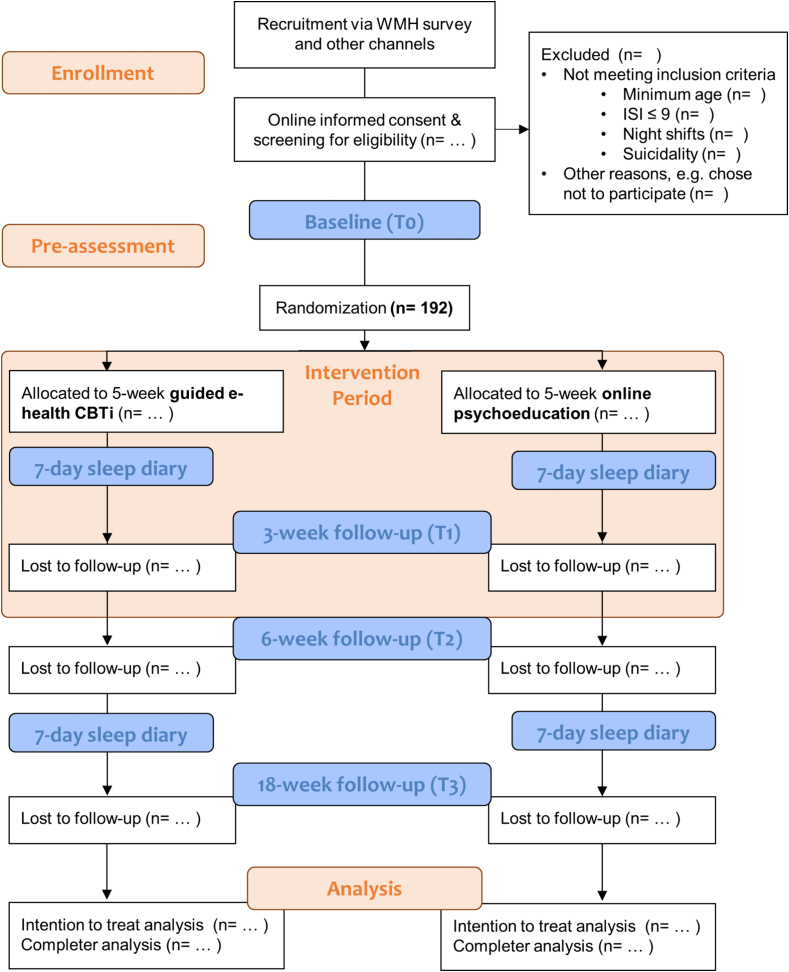
Study design and participant flow.

This study is a two-arm parallel group superiority randomized controlled trial (RCT) with repeated measures, comparing the guided online *‘i-Sleep & BioClock’* intervention to online sleep hygiene and psychoeducation. This RCT follows a previously conducted open pilot study which was conducted from May to December 2022. The pilot study aimed to test the feasibility, acceptability, and preliminary effectiveness of the intervention. Users' feedback has been incorporated and indications for effectiveness have been found. Participants will be randomized in blocks to either condition in 1:1 ratio, stratified by gender and university. The intervention group will receive the 5-week *‘i-Sleep & BioClock’* program, which is an online digital CBT-I intervention enhanced with components on the biological clock. The control group will receive brief online psychoeducation (PE) without any interactive components and without support of the e-coaches. Participants in the control group will be offered the guided online program after 18 weeks. The design of the trial and study flow is shown in Fig. 1. This trial has been approved by the Medical Ethics Committee Leiden Den Haag Delft (NL83395.058.23) on July 17th 2023 and registered at ClinicalTrials.gov (NCT06023693). This protocol has been written in accordance to the SPIRIT guidelines and the checklist is presented in an additional file (Chan et al., 2013). The study will be reported in adherence to the Consolidated Standards of Reporting Trials (CONSORT) statement (Moher et al., 2010).

### 2.2 Participants & setting

The study takes place in a university setting within nine Dutch universities (Leiden University, VU Amsterdam, University of Utrecht, Maastricht University, Erasmus University Rotterdam, Universiteit van Amsterdam, Inholland University of Applied Sciences, Avans University of Applied Sciences, and Rotterdam University of Applied Sciences). These universities belong to the Caring Universities Consortium, the Dutch branch of the World Health Organization College Student Initiative (The World Mental Health Survey Initiative, 2023), who conduct a yearly mental health screening in students and offer free online services to improve student's mental wellbeing.

Students are eligible when they fulfil the following criteria: having self-reported insomnia symptoms of at least subthreshold clinical severity (Insomnia Severity Index-score ≥ 10; ISI) (Morin et al., 2011), aged 16 years or above, affiliated with one of the participating Universities as Bachelor, Master or PhD student, being fluent in English or Dutch. Exclusion criteria are a current risk for suicide. Students are asked about the occurrence of suicidal thoughts in the past 12 months. After positive response, they are asked what the likelihood of acting on the suicidal thoughts is in the coming 12 months. If the likelihood of acting on these thoughts is anything other than “Not likely at all”, they are excluded from the study. The second exclusion criteria are night shifts (not being able to comply with the intervention due to night shifts, meaning work between 2 AM and 6 AM at least once a week). Due to the pragmatic nature of the trial, students using medication or receiving additional psychological treatment will not be excluded as they might still benefit from the intervention, but use of additional treatments will be monitored.

### 2.3 Recruitment & randomization

Students will be recruited via social media, email newsletters, advertisements, and student support services at the participating universities. Furthermore, a mental health screening survey will be sent out annually to students of all participating universities in order to identify students with psychological problems. Students who fulfil inclusion criteria will be invited to follow the *‘i-Sleep & BioClock’* program. Students will use their student email address to start the registration procedure. First, they receive information regarding the study and the program via a detailed ‘landing-page’, and provide digital informed consent (in case of students aged ≤18 years, the parent or legal caregiver will provide informed consent). After account activation and successful completion of the baseline questionnaires, participants will be automatically randomized into either one of the groups, in 1:1 ratio. An independent person not connected to this study will produce a computer-generated randomization scheme stratified by university and gender. Randomization will take place with block randomization, using twenty blocks of six allocations. The allocation is concealed from the researchers. The researchers will not allocate or inform participants about their condition themselves as the randomization is implemented on the platform and is fully automated. Participants, coaches, and researchers cannot be blind for allocation to conditions. However, the researcher will be blinded for the analysis. The intervention group will follow the *‘i-Sleep & BioClock’* intervention and the control group will follow the online PE.

### 2.4 Intervention

The ‘*i*-*Sleep & BioClock*’ intervention for students is largely based on the i-Sleep intervention which was developed by Prof. dr. Annemieke van Straten and Dr. Jaap Lancee (van der Zweerde et al., 2019a). The i-Sleep intervention was developed based on the existing literature and uses evidence-based techniques that are commonly incorporated in face-to-face CBT-I. The intervention aims to reduce insomnia symptoms. The effectiveness of this intervention has been established in patients with insomnia (Van Der Zweerde et al., 2016). The life of a university student typically involves late-night-studying, social activities, part-time-jobs and other factors leading to irregular sleep schedules. University students might therefore be more vulnerable to disturbances of the day and night rhythms than other young adults with regular job or school hours. Learning about and understanding circadian rhythms might help students to improve their sleep and overall wellbeing. Therefore, we adapted i-Sleep to the specific needs of university students and added elements of the biological clock (e.g. light exposure, social jetlag, and chronotype assessment). Furthermore, we adjusted the language to a more informal style and added more interactive and engaging elements. The new version of the intervention was named *‘i-Sleep & BioClock’* and it consists of five modules: (Jiang et al., 2015) psychoeducation on insomnia, the biological clock, and sleep hygiene; (Lund et al., 2010) stimulus control and sleep restriction; (Van Den Berg et al., 2018) worrying and relaxation; (Taylor Daniel, 2012) cognitive interventions to change dysfunctional thoughts about sleep; and (Hershner and Chervin, 2014) summary module, relapse prevention, and plan for the future.

The modules consist of texts, question boxes, exercises, quizzes, memes, gifs, interactive elements (flip-cards, multiple choice questions with automated feedback), and audio-visual elements (e.g. explanatory videos and relaxation audios). Each module is designed to last for about 30 to 60 min and it is recommended to complete one module per week. Therefore, the duration of the *‘i-Sleep & BioClock’* intervention will be approximately five weeks, although students can follow the program at their own pace. In between modules, the student is given exercises (e.g. adapting the sleep pattern, practising relaxation exercises). In addition, students are asked to keep a daily record of their sleep and light exposure habits in form of a diary. They will optionally receive daily reminders via SMS or email for this diary. The program is available in both Dutch and English and can be accessed via any web browser, also via web browsers on smartphones. An overview of the intervention content can be found in Table 1.

**Table 1:** Content of the i-Sleep & BioClock intervention.

Intervention content	
Module	Content
Module 1	Psychoeducation about sleep and insomnia, psychoeducation on the biological clock: the circadian rhythm, chronotypes, importance of light behaviour Basic sleep hygiene: information about behaviours that are known to promote or impede sleep e.g. performing physical exercise or the use of caffeine
Module 2	Sleep restriction and stimulus control: patients are taught to use the bedroom only to sleep and to restrict the time in bed to the average amount of night-time sleep
Module 3	Worrying and relaxation: audio files with progressive muscle relaxation exercises are offered and techniques to stop worrying (e.g. thought blocking, evaluation and worry time)
Module 4	Erroneous cognitions about sleep: the basics of cognitive behavioural therapy are explained and the most common erroneous ideas about insomnia are discussed
Module 5	Summary and plan for the future

#### 2.4.1 Coaching

Guidance in digital interventions can enhance the benefits of internet-delivered CBT-I and encourage more participants to complete the intervention (Lancee et al., 2013). Participants will receive weekly personal feedback on exercises, diary, and personal progress from online coaches via a chat messenger on the Caring Universities platform. *E*-coaches will be trained third-year Bachelor level or Master level clinical psychology students. These coaches will be trained and supervised by a psychologist. Participants in the intervention group will receive an intake call from their e-coach within two days after registration, discussing the coaching structure, individual goals, the sleep and light exposure diary, and study measurements. The call is furthermore aimed at establishing a personal connection to the coach, increasing commitment to the program, and preventing dropouts. In the registration process, students can choose to sign up anonymously and their name will be hidden from their coach. These anonymous students will not receive any calls from their coach but will instead be contacted by the researcher for this call. *E*-coaches will provide asynchronous written personalized feedback to each participant through the program platform within 72 h after session completion. The aim of the written feedback is to increase motivation and adherence of the participants. E-coaches will send personal reminders to participants who have not been on the platform for more than two weeks. In case of adverse events or crisis situations, e-coaches will report to their supervisor and follow the appropriate protocols.

### 2.5 Control group – online psychoeducation

The control group will receive access to the platform and the sleep and light exposure diary. The control condition receives the *‘Sleep Well’* program, which is brief online PE for insomnia and is provided within the same time frame as the intervention in the active condition (between T0 and T2). It involves a comprehensive online psychoeducation module on sleep hygiene, providing detailed information on maintaining a regular sleep schedule, creating a restful sleep environment, and understanding common sleep issues. Although the contact time and content delivery differ from the intervention group, the psychoeducation module is extensive and designed to offer meaningful and beneficial information. Psychoeducation as a control condition was chosen because it can enhance the external validity of the study by simulating a more realistic comparison group than to waitlist or no intervention. Both conditions have access to their program for a total of two years. The online PE is similar to module 1 of the active condition and contains recognized sleep hygiene advice, for example, recommendations about evening routines, and use of alcohol and caffeine. Students will be advised to monitor their sleep in the diary. Key differences to the *‘i-Sleep & BioClock’* intervention are that the online PE (Jiang et al., 2015) does not provide individualized support by an e-coach; (Lund et al., 2010) includes significantly simplified, less detailed content with no interactive components, and (Van Den Berg et al., 2018) is not delivered in a step-by-step manner, but will be provided all at once. The control group will be offered the intervention with coaching after the final measurement at 18 weeks. They are also able to access regular health care services if needed.

### 2.6 Outcome measures

The outcomes will be evaluated at baseline (T0), 6 weeks (T2), and 18 weeks (T3) after randomization. The T2 measurement is the primary endpoint. Additionally, insomnia severity and the two cognitive mediator variables (DBAS-10 and PSAS) will have an extra assessment point at 3 weeks (T1) after randomization. The questionnaires will be sent out via email and will be filled out in a secured online environment. Participants will be compensated for completing the T2 questionnaires (€15,- voucher) and T3 questionnaires (raffle of eight vouchers of €50,-). Table 2 shows an overview of the assessment instruments used for each time point.

**Table 2:**
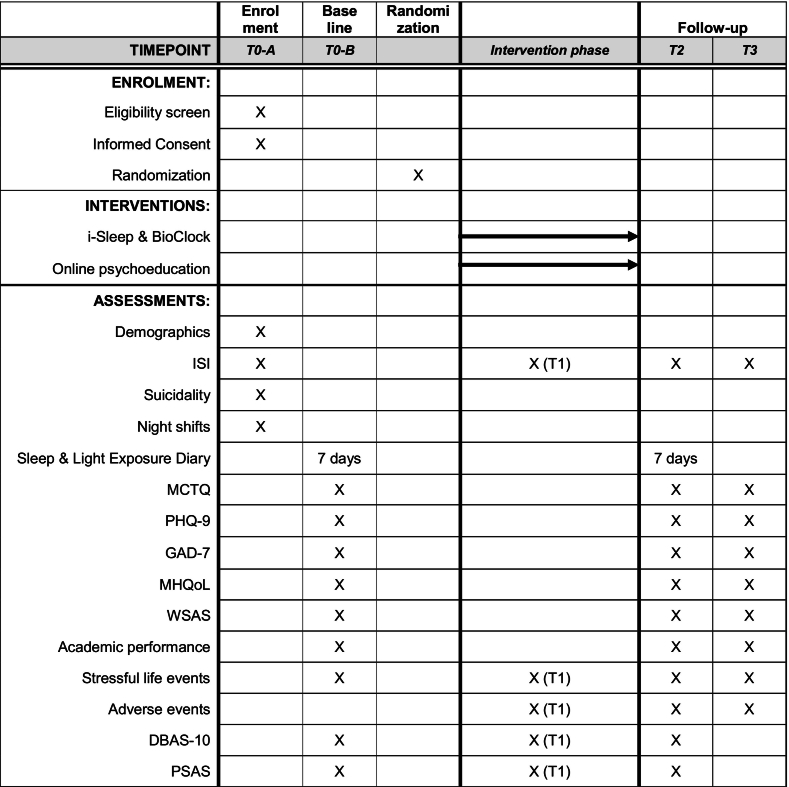
Overview of the assessment moments and their content.

*Note*. T0-A: Eligibility screening a moment of registration; T0-B: Baseline measurement at moment of registration; Intervention phase: 5-week intervention plus one week margin; T1: Mid-treatment measurement at end of week 3; T2: Post-test 6 weeks after baseline; T3: Follow-up 18 weeks after baseline; ISI: Insomnia Severity Index; MCTQ: Munich Chronotype Questionnaire; PHQ-9: Patient Health Questionnaire; GAD-7: Generalized Anxiety Disorder Scale; MHQoL: Mental Health Quality of Life Questionnaire; WSAS: Work and Social Adjustment Scale; DBAS: Dysfunctional beliefs and attitudes about sleep; PSAS: Pre-Sleep Arousal Scale.

#### 2.6.1 Primary outcome

The primary outcome will be *insomnia severity*, measured with the Insomnia Severity Index (ISI), which is a reliable and valid instrument, with internal consistency coefficients of *α* = 0.90 in community samples and *α* = 0.91 in clinical samples (Morin et al., 2011). The ISI consists of 7 items with a 5-point Likert scale (0 = not at all, 4 = extremely) and scores range from 0 to 28, with higher scores indicating more severe insomnia. The ISI has been validated for online use (Thorndike et al., 2011). The main endpoint will be at post-test (6 weeks after baseline). Secondary endpoint will be the 18-week follow-up. Participants are treatment remitters if the ISI score at T2 is <10, and treatment responders if there is a reduction in the total ISI score of >8 (Morin et al., 2011).

#### 2.6.2 Secondary outcomes

Secondary outcomes will be administered at baseline (T0), 6 weeks (T2), and 18 weeks follow-up (T3).

##### 2.6.2.1 Munich Chronotype Questionnaire (MCTQ)

We will assess the 17-item MCTQ, which is a relatively objective self-report questionnaire, since it deals with specific sleep- and wake times. The MCTQ asks for sleep- and wake-times on work/study days and on free days over the past four weeks. Based on this, we can calculate *social jetlag* (the difference in mid-sleep between work/study days and free days), *average weekly sleep duration*, and *average weekly light exposure*, which will be reported as secondary outcomes. We can also determine the *chronotype* (the midpoint between sleep on- and offset on free days corrected for sleep debt accumulated over the workweek), which will be assessed as a moderator (Roenneberg et al., 2003). The MCTQ mid-sleep point shows high correlation with dim light melatonin onset, which is the gold standard for assessing central circadian timing in humans (Kantermann et al., 2015).

#### 2.6.2.2 Patient Health Questionnaire (PHQ-9)

*Depressive symptoms* will be measured with the 9-item PHQ-9 (Kroenke et al., 2001), which is a frequently used, reliable and valid measure for severity of depression (Wittkampf et al., 2007). The items are on a scale from 0 to 3 with total scores ranging from 0 to 27. Higher scores indicate higher depression severity.

##### 2.6.3.1 Generalized Anxiety Disorder Scale (GAD-7)

*Anxiety symptoms* are measured with the 7-item GAD-7 (Spitzer et al., 2006). The items are on a scale from 0 to 3 with total scores ranging from 0 to 21. Higher scores indicate higher anxiety severity. The GAD-7 is a psychometrically sound measure in university students (Byrd-Bredbenner et al., 2020).

##### 2.6.3.2 Work and Social Adjustment Scale (WSAS)

*Daily functioning* will be assessed with the 5-item WSAS, a brief and reliable 5-item questionnaire to assess impairment of functioning (Mundt et al., 2002). Items have a scale from 0 to 8 with total scores ranging from 0 to 40. Higher scores indicate more impairment in functioning. The WSAS shows high internal reliability and sensitivity to treatment effects (Zahra et al., 2014).

##### 2.6.2.5 Mental Health Quality of Life Questionnaire (MHQoL)

*Quality of life* will be measured with the 8-item MHQoL, which is a simple and effective measure to assess quality of life (Van Krugten et al., 2021). It has seven dimensions, covering self-image, independence, mood, relationships, daily activities, physical health, and future, as well as a general visual analogue scale. Items are answered on a four-level scale, and scores are ranging from 0 to 21, with higher scores indicating a better quality of life. The MHQoL-VAS visual scale additionally measures general psychological well-being on a scale of 1–10.

##### 2.6.2.6 Academic performance

Students will be asked about the grade of their last exam (Grade 1–10), the average grade of the past semester (Grade 1–10), whether they failed any courses in the past semester (Yes/No), and their study progression in the past semester in terms of ECTS achieved (30 ECTS per semester: Yes/No). The grading system is consistent across universities.

##### 2.6.2.7 Sleep & light exposure diary

The sleep and light exposure diary will be assessed in the first seven days after baseline measurement (week 1) and in the seven days after the post-test measurement (week 7). The sleep diary is based on the Carney consensus sleep diary and the light exposure items on the Harvard Light Exposure Assessment (Carney et al., 2012; Bajaj et al., 2011). Text adaptations were made in consensus with a group of sleep researchers. Outcomes that will be derived from this diary will be *sleep onset latency* [SOL; number of minutes from lights off to sleep onset], *wake after sleep onset* [WASO; number of minutes awake between sleep onset and final awakening], *early morning awakening* [EMA; number of minutes between final awakening and getting up], *number of awakenings* [NA], *time in bed* [TIB; number of minutes between lights off and getting up], *total sleep time* [TST; TST = TIB – SOL – WASO - EMA], *sleep efficiency* [SE; SE = TST / TIB x 100]. Students also rate their *sleep quality* and how *rested* they feel in the morning [both rated from 0 to 10]. Additionally, *timing and duration of light exposure* and *screen use before bedtime* [Yes/No] will be assessed. Diary items are described in detail in a supplementary file.

#### 2.6.3 Other measures

##### 2.6.2.3 Dysfunctional beliefs and attitudes about sleep (DBAS-10)

We will measure *dysfunctional beliefs and attitudes about sleep* with the abbreviated DBAS-10 (Edinger and Wohlgemuth, 2001). The 10 items are scored on a scale from 0 (strongly disagree) to 10 (strongly agree). The DBAS-10 has appropriate internal consistency, effectively discriminates normal sleepers from insomnia sufferers, and detects cognitive changes resulting specifically from CBT interventions (Edinger and Wohlgemuth, 2001).

##### 2.6.2.4 Pre-Sleep Arousal Scale (PSAS)

We will measure *pre*-*sleep arousal* with the 16-item PSAS. The items are scored on a 5-point Likert Scale, with higher scores reflecting more arousal. The PSAS is a commonly used scale and seems to have appropriate psychometric properties (Jansson-Fröjmark and Norell-Clarke, 2012).

##### 2.6.3.3 Stressful life events

Stressful life events can have an impact on sleep and will therefore be examined as a possible covariate (Li et al., 2019). We will ask about whether participants experienced life-threatening or serious accident/ illness, death of someone close, physical/sexual violence or abuse or other incidents, at all time-points.

##### 2.6.3.4 Adverse events

Adverse events will be examined at T1, T2, and T3, asking about 1) falling accidents, 2) traffic accidents, or 3) any other accidents related to fatigue or sleepiness, and its consequences. Clinical symptom deterioration will also be reported based on the reliability change index (Jacobson and Truax, 1991).

##### 2.6.3.5 Adherence

The main indicator for adherence to the program will be the number of modules completed. The number of modules completed at the time of T2 divided by the total number of modules and multiplied by 100 will be reported. Furthermore, we will report the number of logins to the platform and the time spent on the platform (in minutes).

### 2.7 Sample size

A previous systematic review for digital CBT-I interventions in adults showed effect sizes on various sleep measures ranging from *g* = 0.21–1.09 (Zachariae et al., 2016). A recent systematic review and meta-analysis showed a moderate effect of sleep interventions compared to both active and passive control groups in reducing sleep disturbance in university students, with an SMD of −0.55 [CI: −0.84, −0.26] at post-test (Chandler et al., 2022). However, given our active control condition and pragmatic design, we anticipate a smaller effect size of Cohen's *d* = 0.4.

Sample size calculations for mixed effect models require a number of estimates, or assumptions (Twisk, 2013). We estimate the (within-subject) intraclass correlation coefficient (ICC) of the baseline assessment and three follow-up assessments to be approximately 0.23. A somewhat higher ICC will slightly inflate the minimal detectable effects, a somewhat lower ICC will slightly lower the minimal detectable effects. At a significance threshold of *α* = 0.05 and a power 1-*β* = 0.80, we estimate that a number of 48 participants per group is needed to detect a small to medium effect size of *d* = 0.4. Given the estimated high drop-out rate of 50 % (Zachariae et al., 2016), we plan to include 192 participants in total.

### 2.8 Statistical analysis plan

All statistical analyses will be conducted in the R statistical programming package. All analyses will be performed according to the intention-to-treat approach, meaning all participants who are randomized will be included in the analyses regardless of study completion. Additionally, we will perform a completer-analysis with only those participants who completed the post-test and follow-up assessments, and a dose-response-analysis with adherence to the modules as a predictor of the intervention effect on insomnia severity. We will apply a two-tailed significance level of *α* = 0.05. Assumptions underlying the planned analyses, such as normality and constant variance of residuals, will be checked, reported, and addressed.

#### 2.8.1 Effectiveness analyses

We will fit linear mixed models to examine the primary outcome of change in ISI score between baseline and post-test assessment. We will estimate the effects of time and the interaction between time and group allocation. Due to the repeated measures, the observations are structured hierarchically within the participants. We are primarily interested in the effect of the intervention from baseline to post-test (T0-T2), but we will investigate in several post-hoc analyses whether the effect was maintained between T2 and T3. The independent variable will be group allocation (to either *‘i-Sleep & BioClock’* intervention or online psychoeducation) and the dependent variable will be insomnia severity (ISI). We will investigate demographics (e.g. age, sex, year of education, marital status, living situation), baseline severity of symptoms, and stressful life events as covariates, in case of differences at baseline. Clinical significant treatment response from T0-T2 will be reported, defined as reduction in total ISI score of ≥8 points (Morin et al., 2011). Furthermore, we will calculate the effect size Cohen's d by taking the mean difference of the outcomes between intervention and control group and dividing the results by the pooled standard deviations. Cohen's d can be interpreted small <0.2, medium around 0.5, or large >0.8 (Cohen, 1988). For the secondary outcomes (MCTQ, PHQ-9, GAD-7, WSAS, MHQoL and sleep & light diary outcomes), we will follow the same statistical approach as for the primary outcome measure. Negative binominal mixed models will be used for skewed sleep & light exposure diary data with excess zeros (Green, 2021).

#### 2.8.2 Mediation and moderation analyses

Mediator and moderator analyses are exploratory and follow the principles by Kraemer et al. (Kraemer et al., 2002). Chronotype will be examined as moderator of the intervention effect on insomnia, by adding the variable to the linear mixed models of insomnia severity as an interaction term (e.g. time x group allocation x moderator). If chronotype will have a significant interactive effect with treatment on the outcome, it is a moderator; with main effect and no interactive effect it is a predictor of response (Kraemer et al., 2002). Additionally, an exploratory investigation of the potential moderating variables will be conducted, including sociodemographic variables and symptom severity, without pre-defined hypotheses, by using the QUINT method (Qualitative Interaction Trees) (Dusseldorp et al., 2016a). The QUINT method identifies three participant subgroups based on baseline characteristics: one where the intervention is more effective, one where the control is more effective, and an optional third where both are equally effective. It includes multiple moderators simultaneously without predefined interactions, detecting higher-order interactions, and represents results as an easy-to-interpret binary tree (Dusseldorp et al., 2016b). Outcome expectations, adherence, and stressful life events will be tested as predictors of treatment response. Furthermore, predictors of adherence, such as demographic variables, symptom severity, and stressful life events, will be investigated.

Light exposure, circadian shift, variability in sleep patterns, pre-sleep arousal, and dysfunctional beliefs and attitudes about sleep will be evaluated as potential mediators of the intervention effect on insomnia severity. The mediators are measured at three time points (T0, T1, and T2), allowing to examine whether treatment-induced changes in mediating variables at T1 predict subsequent changes in outcome at T2. Mediation analysis will be conducted by using multilevel structural equation modeling in R (Preacher et al., 2010), with insomnia severity as dependent variable (Y), group allocation as independent variable (X), and the potential mediator variables (M). All mediators will be investigated separately first and significant mediators will be included in a combined model.

## 3 Discussion

Sleep and mental health problems are highly prevalent among university students (Auerbach et al., 2018). They are impairing students' functioning, wellbeing, and quality of life, and can have far reaching long-term consequences (Baglioni et al., 2011). There is a great need for easily accessible and effective interventions to treat insomnia and the related psychological problems among students. In the adult population, internet-based CBT-I has already been proven to be beneficial for improving sleep other mental health complaints (Espie et al., 2019; Zachariae et al., 2016; van der Zweerde et al., 2019b). In the population of university students the research is still more scarce.

This study protocol presents the design of a randomized-controlled trail, to investigate the effectiveness of a guided e-health intervention *‘i-Sleep & BioClock’* in university students. The primary aim of this study is to investigate whether such an intervention can help students to overcome their insomnia complaints. The secondary aim is to investigate whether mental health symptoms improve due to the intervention and whether the effects are maintained in the longer term. Thirdly, moderators, mediators, and predictors of response will be evaluated in order to understand how and for whom the intervention works.

The current study shows several strengths. Firstly, this is a pragmatic randomized controlled trial because it is conducted in a real-life setting, improving its external validity and making the results generalizable. Secondly, a previous pilot study has been conducted to minimize errors in the trial. Thirdly, the analysis will follow the intention-to-treat approach, which is a method known to reduce bias and provide a realistic estimation of the effectiveness. Lastly, *‘i-Sleep & BioClock’* is a low-threshold intervention, as it is easily accessible from any location and device with internet connection, at any time without the constraints of a formal intake or appointment, office hours or therapist schedules, and the possibility to register anonymously and receive help without having to fear stigma. It requires less resources than traditional CBT-I and in case it is effective it could be implemented immediately on a larger scale. Additionally, several potential limitations of the study should be noted. Firstly, our last follow-up moment is at 4.5 months after baseline, making it impossible to draw conclusions about the long-term effects of the intervention. Secondly, despite the fact that we examine many different outcome measure, all of those are self-report measures. Thirdly, all contact with participants is online or via the phone, which might lead to higher drop-out than in face-to-face interventions. Nevertheless, this approach is most fitting to the low-threshold characteristic of the intervention and moreover it minimizes burden for participants. High drop-out rates are common in e-health interventions and we indeed expect a high drop-out rate, where data might not be missing at random. However, we will implement different strategies to enhance completion of the intervention and measurements, such as phone calls by the e-coach, reminders, and monetary incentives to complete the questionnaires. Lastly, the exclusion of students with suicidal ideation limits the generalizability of the findings.

Preventing exacerbation of mental health problems among students is a very important step toward reducing the burden of mental health problems among this vulnerable group. Early interventions should be prioritized to avoid the manifestation of more severe mental health conditions. In case the online self-help intervention is effective in university students, this might encourage the dissemination of the *‘i-Sleep & BioClock’* intervention in universities worldwide.

## Abbreviations

Cognitive Behavioural Therapy for Insomnia: CBT-I
Early morning awakening: EMA
Generalized Anxiety Disorder Scale: GAD-7
Dysfunctional beliefs and attitudes about sleep: DBAS-10
Duration of Sleep Episode: DSE
Insomnia Severity Index: ISI
Munich Chronotype Questionnaire: MCTQ
Mental Health Quality of Life Questionnaire: MHQoL
Number of awakenings: NA
Patient Health Questionnaire: PHQ-9
Psychoeducation: PE
Randomized controlled trial: RCT
Pre-Sleep Arousal Scale: PSAS
Sleep efficiency: SE
Sleep onset latency: SOL
Time in bed: TIB
Total sleep time: TST
Wake after sleep onset: WASO
Work and Social Adjustment Scale: WSAS

## Trial status

Start of recruitment: November 2023.

End of recruitment: planned for July 2024.

## Ethics approval and consent to participate

The study protocol was approved by the METC Leiden Den Haag Delft on July 17th 2023 (NL83395.058.23). Written digital informed consent to participate will be obtained from all participants.

## Consent for publication

Not applicable.

## Funding

The study is part of the BioClock Consortium and is funded by the Dutch Research Council (10.13039/501100003246Nederlandse Organisatie voor Wetenschappelijk Onderzoek; NWO), with grant number: NWA.1292.19.077. The Dutch Research Council is not involved in data collection, analyses and interpretation of the data nor in writing the manuscripts.

## CRediT authorship contribution statement

NA is the principal investigator and wrote the study outline for the grant application. All authors contributed to the study design: LP, NA, AvS, SS, and PS. The protocol paper was written by LP. All authors contributed to the drafting of the submitted version of the study protocol and all authors approved the final version of manuscript.

AvS developed the initial i-Sleep intervention. This initial version is commercially available and AvS receives a small return on investment to be invested in new research projects. For the current project, i-Sleep was adapted for students by LP and NA, with input from all authors. This version is only available for research purposes. There are no other commercial interests. The remaining authors declare that they have no known competing financial interests or personal relationships that could have appeared to influence the work reported in this paper.

## Declaration of competing interest

AvS developed the initial i-Sleep intervention, which has been further developed by LP and NA, with input by all authors. None of the authors have any commercial interest. The authors declare that they have no known competing financial interests or personal relationships that could have appeared to influence the work reported in this paper.

## Data Availability

The datasets generated and analysed during the current study are available from the corresponding author on reasonable request, and in the DANS repository after study completion.

## Appendix A Supplementary data

Supplementary material Image 1
