# Supplementary material for: Effectiveness of a guided digital self-help intervention to improve sleep and the biological clock in university students – Study protocol for a randomized controlled trial
Source: Internet Interv. 2024 Aug 2;37:100763. doi: 10.1016/j.invent.2024.100763 (PMC11367106; doi:10.1016/j.invent.2024.100763)
Supplement: Supplementary file 1 — Supplementary material [file mmc1.docx]

| **#** | **Item** | **Question and explanation** |
| --- | --- | --- |
| 1 | Time of lights out | What time did you go to sleep?  This concerns the night of  Sunday (dd-mm-yyyy) to Monday (dd-mm-yyyy)  Explanation: Please indicate what time you went to sleep. This is the time when you turned off the light to go to sleep.  Example 1: you went to bed at 11:00 PM and turned the light off immediately. In this case you write down 11:00 PM.  Example 2: you went to bed but read for half an hour and turned off the lights at 11:30 PM. In this case you write down 11:30 PM.  Example 3: you went to bed and turned off the light at 23:00 PM but ended up falling asleep only at 01:00 AM. In this case you write 23:00. You can indicate the 2 hours of lying awake on the next page. |
| 2 | Time of getting up | What time did you get up?  This concerns the night of  Sunday (dd-mm-yyyy) to Monday (dd-mm-yyyy)  Explanation: Indicate here what time you got out of bed. This may be a different time than the time you woke up.  Maybe you woke up at 7:30 AM but got up at 8:00 AM. In this case, choose 8:00 AM here and continue on the next page. |
| 3 | Periods of being awake between time of lights out and time of getting up | Have you been lying awake?  Indicate when you were awake on the night of  Sunday (dd-mm-yyyy) to Monday (dd-mm-yyyy)  Explanation: Indicate here when you were awake during the night. You can also choose whether you went out of bed during the time you were awake at night or whether you stayed in bed. Select the boxes from the timetable by clicking. One box equals 15 minutes. |
| 4 | Times of napping | Did you take a nap?  Indicate when you took a nap on  Sunday (dd-mm-yyyy)  Explanation: A nap is the amount of time you slept during the day, whether you were in bed or not. Select the boxes from the timetable by clicking. One box equals 15 minutes.  You did not take a nap? Then skip this page and click on 'Next'. |
| 5 | Times of being outside | Have you been outside?  Indicate when you went outside on  Sunday (dd-mm-yyyy)  Explanation: Indicate when you have been outside the house. These are all the moments when you have not had a roof over your head.  You have not been outside? Then skip this page and click on 'Next'. |
| 6 | Screen use before sleeping | Screen use before sleeping?  This concerns the evening of  Sunday (dd-mm-yyyy)  Yes/No  Explanation: Did you use a screen an hour before going to sleep (such as a mobile phone, tablet, e-reader, laptop, or TV)? Only indicate "Yes" if you have looked at the screen for at least 5 minutes. Indicate "No" if you have only set an alarm clock. |
| 7 | Feeling of being rested | How rested do you feel this morning?  This concerns the night of  Sunday (dd-mm-yyyy) to Monday (dd-mm-yyyy)  Scale of 0 (not at all rested) to 10 (very much rested) |
| 8 | Sleep quality | How would you rate the quality of your sleep?  This concerns the night of  Sunday (dd-mm-yyyy) to Monday (dd-mm-yyyy)  Scale of 0 (very badly) to 10 (very well)  Explanation: Quality of sleep is your own feeling of whether you have slept well or badly. |
